# Grand challenges in chemical engineering

**DOI:** 10.3389/fchem.2014.00017

**Published:** 2014-04-09

**Authors:** Gil Garnier

**Affiliations:** Department of Chemical Engineering, Monash UniversityClayton, VIC, Australia

**Keywords:** grand challenges, chemical engineering, materials and nanotechnology, biomedicine, energy metabolism, green chemistry technology, separation

Chemical Engineering—also referred to as process engineering—is the branch of engineering applying physical and life sciences, mathematics and economics to the production and transformation of chemicals, energy and materials. Traditionally, it consists of heat, mass and momentum transport, kinetics and reaction engineering, chemical thermodynamics, control and dynamic simulation, separation, and unit operations. Conventionally developed and applied for the petro-chemical and the heavy chemical industry, chemical engineering has rapidly evolved with applications in a multitude of fields, including climate change, environmental systems, biomedical, new materials and complex systems.

In 2003, the report “Beyond molecular frontiers: challenges for chemistry sciences and chemical engineering” mandated by the National Research Council of the American National Academies and chaired by Professors Breslow and Tirrell was released (National Research Council, 2003). The study investigated the status of chemical science: where are we, how did we arrive at this state and where are we heading? It concluded that science has become increasingly interdisciplinary. It also identified a trend toward the strong integration from the molecular level to chemical engineering and “*the emergence of the intersections of the chemical science with all the natural sciences, agriculture, environmental science and medicine as well as with materials science, physics, information technology and many other fields of engineering*.” A decade later, this vision has been largely realized and so-called “molecular engineering” that integrates chemical engineering with all sciences is now a reality. These rapidly expanding intersections of a wide range of areas of science with engineering are the new Frontiers in Chemical Engineering.

Frontiers in Science and Engineering are mobile, ever expanding in a non-linear and stochastic fashion. Any attempt to map the frontiers of knowledge is a difficult exercise that is usually out of date before it is published. An arguably more profitable alternative is to challenge the frontiers: to push their boundaries until some reaction occurs: whether rejection by the community or some progress follows in incremental or quantum steps.

Another approach to define the frontiers of chemical engineering is to consider the chemical reactions that have marked the development of humanity's current standards of living and the topics currently critical to ensure that acceptable standards are distributed more equitably around the globe without catastrophic impact on global climate and ecosystems. What is the most important chemical reaction that has impacted humanity? And what will be the next one? What are the most significant chemical technologies needed to ensure expansion of acceptable living standards while minimizing environmental impact?

To take just one of many possible candidates for the title of “Most Important Chemical Process,” the Haber-Bosch reaction, which produces ammonia by reacting atmospheric nitrogen with hydrogen, has allowed humanity to pass the 2 billion population barrier and reach the current global population of some 7 billion (Smil, 1999; Kolbert, 2013). Ammonia is a key ingredient in fertilizer for good plant growth. Until the advent of the Haber-Bosh process in the 1913, agriculture operated under nitrogen-limited conditions with the cultivation of arable lands sufficient to feed only 2 billion people. Developing low cost fertilizer has enabled a new era of growth in both crop yields and human nutritional standards by escaping the limitations imposed by natural nitrogen fixation processes. An agricultural revolution has been the result.

Another example of chemical processes with wide social significance are the development of antibiotics, vaccines and immunology which have given mankind much better control over microbial pathogens, allowing longer and better human lives. Yet a third area of chemistry is our understanding of semiconductor materials and how to mass produce them with extraordinary precision that is the basis of modern microelectronics, computer science and the World Wide Web. These chemical and electronic technologies have effectively decoupled the memory/storage function of the human brain from its analytical capability, thereby liberating its powers to focus on creativity and connectivity in ways that previous generations could not imagine. Increasingly sophisticated application of mathematical principles to the phenomena of physics, chemistry and biological sciences, from the atomic level to intergalactic scales, enable us to better understand natural and anthropogenic phenomena and to either control them, or to prepare for changes which are beyond our control.

Langer and Tirrell, from MIT and Caltech respectively, have pioneered an engineering approach to biomaterials for medical application, even pushing the boundary of oncology and tissue engineering (Langer and Tirrell, 2004; Karp and Langer, 2011; Schroeder et al., 2011). Bird et al. showed that molecular engineering of surface affects not only the behavior of liquid droplets with a surface at equilibrium, but also their dynamic interaction (Bird et al., 2013).

When addressing industrial and practical problems, we often also challenge frontiers in chemical engineering. Chemical engineering represents both the application of science and the link between chemistry, society, and industry. Chemical engineering studies often push the boundaries of chemistry by applying model systems and equations developed with well-behaved systems to complex industrial challenges. The engineering approach rates and quantifies the relative importance of combined, antagonistic, or synergistic systems. With the aim of minimizing pitch deposition during papermaking, we recently investigated the effect of salts, shear, and pH on pitch coagulation to discover the effect of ion-specificity and non-ideal behaviors with shear (Lee et al., 2012). In the development of paper diagnostics for blood typing, we quantified the bio-specific reversible coagulation of red blood cells and used adsorption, elution, filtration and chromatography to develop a practical technology. This applied study has highlighted the gap in knowledge on the dynamic interaction of antibodies and macromolecules with surfaces (Khan et al., 2010; Al-Tamimi et al., 2012).

So what are some new frontiers to be challenged? From a multidimensional approach based on field and application they are as follows:

Reaction Engineering

- Combination of organic, inorganic and biochemical catalysis to decrease energy of activation, increase selectivity, reduce energy usage, by-products (separation) and replace toxic organic solvents and reagents based on scarce elements by reactions in aqueous or bio-based solvents using green chemical principles.
- Harnessing photosynthesis to convert solar energy and CO_2_ into glucose, ligno-cellulosic polymers and their intermediates using enzymatic catalysts and/or aqueous systems.
- Understand and optimize mass transfer, energy transfer, extent, and selectivity of reactions in medicine. Applications include the selective destruction of cancer cells, bacteria, fungi, and viruses (infection) and the regulation of immunologic reactions.
- Predictive reaction engineering adjusting rate of reactant and product removal accordingly to kinetics of reaction to minimize side reactions, thereby making separation easier and more efficient.

Unit Operations and Transport Phenomena

- More selective, specific, and low energy separation processes for gas-gas and liquid-liquid systems.
- High flux and anti-fouling reverse osmosis and membrane separations.
- Improved separation of thermally sensitive chemicals having similar boiling points using fractional distillation, or other means.
- Better methods for pumping and transporting suspensions of solids in liquids-especially at high solids contents.

Biomedical

- Develop an engineering approach to model and regulate (control) the behavior and functionality of the human body and mental processes.
- Apply simulation and control strategies to the various hierarchies of biological systems, ranging from DNA and RNA, the cell, tissues, and organs, up to the human body to give improved quality of life to people with genetic and related disorders.
- Minimally invasive sensors to control blood pressure, blood lipid concentrations and heart rate.
- Nanotechnology for selectivity in oncology and drug delivery.
- Biotechnologies and improved biomaterials for organ regeneration.

Energy

- Low cost energy is key to improve living standards for the majority of people in less developed nations. With anthropogenic greenhouse gases causing a slow but steady global warming—an adequately proven reality—a prime challenge is to produce net energy with minimal environmental impact. Chemical engineers have a responsibility to verify and ensure that energy balances and thermodynamics are the best economically achievable. The production of chemicals from renewable source and using green chemistry is an extension of the challenge, and again chemical engineers' incumbent responsibility is to discover processes and reactions with positive thermodynamics and energy balances, then to optimize these processes by active engagement with economists, environmental scientists, and society at large.
- Cost-effective storage of solar energy (including solar energy embodied in wind and ocean currents) to enable distribution at times of peak human demand remains a critical issue. Development of reversible processes for energy storage and utilization that have rapid start-up and shut-down characteristics is therefore of prime importance.
- While rapid and controlled release of large quantities of (mainly) electrical energy is of importance in meeting society's needs, it should not be forgotten that there would be enormous benefit in capturing and storing solar energy in ways that mimic natural photosynthetic processes, so that solar energy is stored in chemical bonds, rather than as heat, or electronic charge separation. If the “artificial” photosynthetic reaction into which the solar energy is “pumped” consumes carbon dioxide, then clearly two major objectives would be achieved in a single technical advance. In this connection it is worth remembering that while the reaction of carbon monoxide with oxygen is highly exothermic, the reverse reaction, namely the thermal dissociation of carbon dioxide into carbon monoxide and oxygen, can occur at the sorts of temperatures that can be reached in a solar furnace (Nigara and Gales, 1986). The remaining technological gaps are development of advanced refractory materials that can withstand the temperatures required to drive the reaction, heat exchange, and efficient separation of the reaction products. Dissolution of carbon monoxide in aqueous alkali to form alkali metal formats would seem to be a promising approach.

Materials

- Multiscale engineering: linking the nano, micro, and meso scales to the macro scale in both materials and processes will be fundamental to the great majority of challenges listed above.
- In order for nanotechnology to advance, molecular engineering using improved molecular dynamic simulations will be essential.
- Use of materials that can be reprocessed into similar products, or if not possible, into a cascade of products of lower value, with the final end-products being completely biodegradable.
- Develop materials and composites from low-energy processes by better understanding of the component structures from the atomic scale to macroscopic properties. Replacement of commodity applications of energy-intensive concrete and metals should be targeted.

Green Chemicals

- The principles of green chemistry have been well publicized (Anastas and Warner, 1998). Maximum use needs to be made of renewable feedstock, utilizing all components. Because biomass has a low energy density compared to fossil carbon sources, the energy efficiencies of biomass processing require critical re-examination, including the development of smaller mobile processing plants that can be taken to the areas where biomass is available on a seasonal basis. Such a re-examination should not exclude possible social and community benefits.
- A key factor in better usage of biomass will be development of new chemical pathways that make more intelligent use of the structures of polysaccharides and lignins. In this connection, the bimolecular mechanisms by which certain insects in the families Hemiptera, and Hymenoptera can manipulate cell differentiation and tissue formation in higher plants to their advantage, by inducing the formation of galls and related, often highly ordered protective structures, made by the host plant certainly warrants detailed multidisciplinary study.
- While a number of useful enzymes are now produced, isolated and used on an industrial scale, the rates at which they catalyze processes are usually limited by thermal instability and denaturation by surfactants and movement of pH outside the neutral range. Chemical engineers have traditionally used heat, pressure, and pH to accelerate chemical reactions, yet the study of the molecular biology of extremophile organisms and their enzymes that have obviously evolved to withstand extreme temperatures, pressures and pH ranges that occur in deep ocean vents and volcanic pools appears to be in its infancy.

Progress in chemical engineering has often been incremental. Initially born of a marriage between mechanical engineering and applied chemistry, chemical engineering has grown into a fully-fledged broad discipline that is constantly seeking new challenges. One area in which many of these challenges are focused improved technologies to harness matter and energy in ways that generate new products, such as organs, energy storage systems, molecularly engineered composites, etc. A closely related area is process optimization to ensure that both existing and new products are manufactured in the most efficient and sustainable ways—in terms of energy and by-products. A third area of challenges is building new facilities and modifying older ones such that they have a clear social license to operate and use the technologies on which society relies to provide acceptable standards of living.

Many of the most interesting and fruitful challenges at the frontiers of chemical engineering involve the integration of chemical engineering with chemistry, physics and biology accompanied by a redefinition of the control volume. In the spirit of this philosophy, the first research topic of Frontiers in Chemical Engineering will be application of chemical engineering principles to oncology with a nanotechnology focus.

## Conflict of interest statement

The author declares that the research was conducted in the absence of any commercial or financial relationships that could be construed as a potential conflict of interest.

## References

[B1] Al-Tamimi M. Shen W. Rania Z. Huy T. Garnier G. (2012). Validation of paper-based assay for rapid blood typing. Anal. Chem. 84, 1661–1668 10.1021/ac202948t 22148716

[B2] Anastas P. T. Warner J. C. (1998). Green Chemistry: Theory and Practice. New York, NY: Oxford University Press

[B3] Bird J. C. Dhiman R. Kwong H.-M. Varanasi K. K. (2013). Reducing contact time of a bouncing drop. Nature 503, 385–388 10.1038/nature12740 24256803

[B5] Karp J. M. Langer R. (2011). Dry solution to a sticky problem. Nature 477, 42–43 10.1038/477042a 21886155 PMC3236562

[B6] Khan M. S. Thouas G. Whyte G. Shen W. Garnier G. (2010). Paper diagnostic for instantaneous blood typing. Anal. Chem. 82, 4158–4164 10.1021/ac100341n 20415489

[B7] Kolbert E. (2013). Fertilizer, fertility and the clash over population growth. The New Yorker 89.33: 96

[B8] Langer R. Tirrell D. A. (2004). Designing materials for biology and medicine. Nature 428, 487–492 10.1038/nature02388 15057821

[B9] Lee R. Lewis T. Richardson D. Stack L. K. Garnier G. (2012). Effect of shear, temperature and pH on the dynamics of salt induced coagulation of wood resin colloids. Colloids Surf. A, 396, 106–114 10.1016/j.colsurfa.2011.12.049

[B4] National Research Council. (2003). Beyond molecular frontiers: challenged for chemistry sciences and chemical engineering. Washington, DC: The National Academies Press

[B10] Nigara Y. Gales B. (1986). Production of carbon monoxide by direct thermal splitting of carbon dioxide at high temperature. Bull. Chem. Soc. Jpn. 59, 1997–2002 10.1246/bcsj.59.1997

[B11] Schroeder A. Heller D. A. Winslow M. M. Dahlman J. E. Pratt G. W. Langer R. (2011). Treating metastatic cancer with nanotechnology. Nat. Rev. Cancer 12, 39–50 10.1038/nrc3180 22193407

[B12] Smil V. (1999). Detonator of the population explosion. Nature 400, 415 10.1038/22672

